# The 2022 Monkeypox Epidemic and What Has Led to the Current State of the Disease in the US: A Systematic Review

**DOI:** 10.7759/cureus.33515

**Published:** 2023-01-08

**Authors:** Samriddh Chaudhari, Leah Treffeisen, Jaswinder Virk, Taral Parikh, Naveen Prasad Gopalakrishnan Ravikumar, Ashish M Goti, Lokesh Goyal, Kanica Yashi

**Affiliations:** 1 Internal Medicine, Kansas City University, Kansas City, USA; 2 Cardiology, Bassett Healthcare Network, Cooperstown, USA; 3 Pediatrics, Hamilton Health Center, Harrisburg, USA; 4 Internal Medicine and Nephrology, Oregon Health and Science University School of Medicine, Portland, USA; 5 Pediatrics, Ochsner Medical Center, New Orleans, USA; 6 Pediatrics, Tulane University School of Medicine, New Orleans, USA; 7 Pediatrics and Child Health, Nice Children Hospital, Surat, IND; 8 Hospital Medicine, CHRISTUS Spohn Hospital Corpus Christi, Corpus Christi, USA; 9 Internal Medicine, Bassett Healthcare Network, Cooperstown, USA

**Keywords:** monkey pox fever, monkey pox virus rash, zoonotic infections, infectious disease control, monkey pox virus

## Abstract

Monkeypox virus (MPOX) is a zoonotic disease in humans. It is similar genetically to its virus family member, smallpox. This virus has been studied since the 1970s. The virus remains endemic to the Congo and West African regions, but non-endemic spreads have been cited. The most recent non-endemic outbreak in the spring of 2022 amidst the current COVID-19 pandemic is of interest due to its impact on global medical, economic, and societal climates.

This literature review aims to highlight the virology, clinical signs and symptoms, diagnosis, prevention, and treatment of MPOX and discuss the social implications of the recent 2022 outbreak.

We hope this review can pinpoint important clinical pearls of the MPOX virus and its societal impacts to further promote important discussion of this virus and its disease.

## Introduction and background

Monkeypox (MPOX) is a smallpox-like viral zoonotic disease caused by an Orthopoxvirus. This virus is an ovoid-shaped double-stranded DNA virus [1,2]. This virus was first noted and studied in central Africa in the 1970s, and its similarities and differences were noted to smallpox clinically and epidemiologically [3]. MPOX virus has been extensively studied since the 1970s outbreak, and the virus is endemic to western and central African countries such as Sierra Leone, the Republic of Congo, Nigeria, Cameroon, etc. [4,5].

There are two clades of the virus, clade 1, Congo basin, originating in Central Africa, such as in Congo, and clade II associating with West Africa. The former is a more transmissible and deadlier virus with a case-fatality ratio of 10%, and the latter is more self-limited with a case-fatality ratio of 3-6% [2]. The virus is spread endemically via animal-human transmission as it is a zoonotic disease. However, non-endemic spreads that have been cited have primarily been via human-to-human contact [2]. The spreads have been cited in the US, UK, Israel, and Singapore since 1970. However, the non-endemic spread that occurred in the spring of 2022 during the already ongoing COVID-19 pandemic is an important event that this review study aims to highlight [6,7].

Methods

Study Setting and Design

A systematic literature review was conducted, and articles were sourced from databases including PubMed, Elsevier, NIH, Google Scholar, Science Direct, and UpToDate, and websites including The United States Center for Disease Control. We searched using keywords including "Monkeypox and the 2022 outbreak," "Monkeypox transmission," Monkeypox pathogenesis," and "Monkeypox in the United States." Each of these sources was searched from the onset of the research for the study to November 20, 2022. 

Inclusion and Exclusion Criteria

Only articles published in English with available abstracts and free access to the full text were included. We excluded commentaries and letters to the editor, expert opinions, unpublished reports, and book chapters. We included systematic reviews, randomized controlled trials, cross-sectional studies, initial cohort, and prospective cohort studies and did not restrict based on the date of publication. We included roughly 500 academic articles in our initial search that comprised data on the diagnosis of MPOX from 1970 to the present. We narrowed down this search to include roughly 100 articles that included our desired data but had a wide cohort of year ranges. We included articles that covered the early years of the initial MPOX outbreaks in the 70s, with articles that included data in subsequent decades instead of early or late cohorts exclusively. We finally excluded articles that did not have detailed symptomatic and treatment information before having a final list to conduct our systematic literature review. The articles that we excluded were solely focused on epidemiology and demographical details of MPOX. In contrast, we included comprehensive articles discussing all aspects of MPOX and its disease, symptoms, and treatments. 

Discussion

Pathogenesis

Infections with MPOX can occur via cutaneous inoculation or respiratory route and are categorized as either systemic or localized. These factors depend on the site of entry and the classification of the virus [8]. Localized disease, limited to the skin and lymphatic system, has been found to result mainly from cutaneous inoculation. Conversely, systemic disease typically results from the respiratory route of infection [8,9]. Whether or not respiratory spread can occur between humans has been unclear, but in the recent 2022 outbreak, there have been reported cases of transmission from prolonged indirect contact [10]. One of the first known cases in the US is presumed to have been from exposure via respiratory droplets on an airplane [11]. It is currently unknown what percentage of cases and in what circumstances MPOX is spread through respiratory secretions [12]. The CDC recommends monitoring for symptoms in patients who have had close indirect contact (secondary exposure) with a known source of infection for over three hours [13]. It is known from animal models that this form of infection has a distinct pathway. The virus starts in the respiratory bronchioles and alveoli after inhalation, travels to nearby lymph nodes, then replicates in the tonsils, spleen, liver, and colon before symptoms arise. Certain groups have been found to be at high risk in the recent outbreak, especially men who have sexual contact with other men [14-16].

## Review

Diagnosis

**Figure 1:**
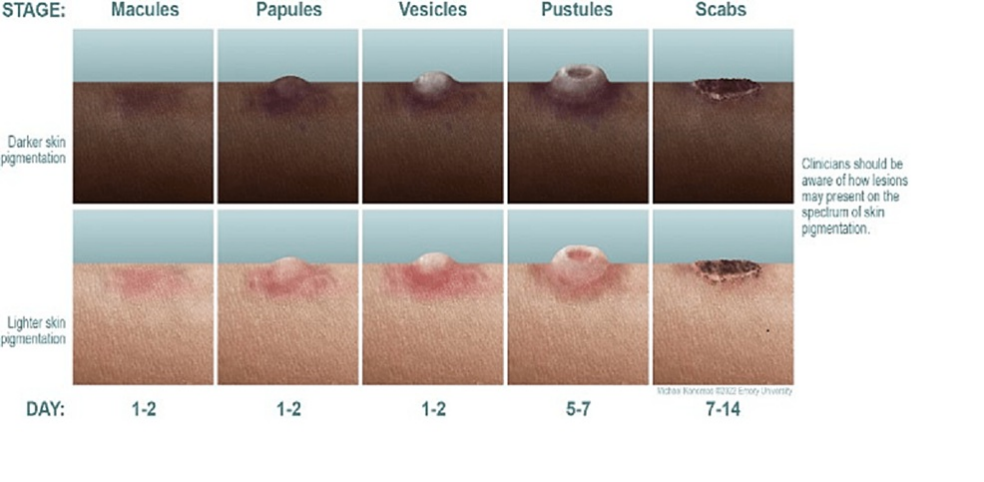
Stages of skin presentation and progression of Monkeypox rash. Source: reference 30

MPOX diagnoses are made via a combination of clinical assessment, patient history, epidemiology, and laboratory findings. This disease has a characteristic rash that often prompts patients to visit their doctor. The typical progression is 2-5 mm macules progressing to papules, vesicles, and finally, pseudo-pustules. This lasts around 1-2 weeks, followed by crusting and flaking off (Figure 1) [17]. Clinical suspicion should be high for MPOX with this rash or in persons with epidemiologic risk factors, such as living in an MPOX endemic region. One example of such a group during the 2022 outbreak is men who have sex with men, as MPOX virus incidence has been higher in this population than in the general population [18,19]. These factors include travel to an area of a recent outbreak, close contact with individuals with suspected or confirmed virus cases, or being part of a community experiencing a higher incidence of the disease. Diagnosis is often confirmed via DNA polymerase chain reaction (PCR) from a swab or biopsy of a skin lesion, but can also be made with serology testing for anti Orthopoxvirus IgM antibody if PCR is not available [17,20].

Clinical features

**Figure 2:**
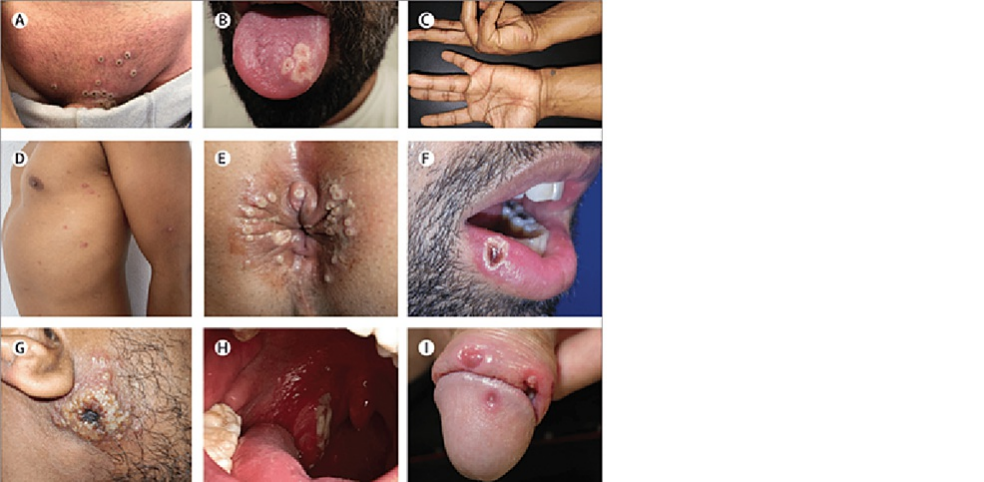
A) Pustules in the genital region. B) Pustular lesions on the left side of the tongue. C) Pearly vesicles in the palmar skin of the hands. D) Scattered papules and pustules with an erythematous halo. E) Pustules in the anal and perianal skin. F) Pustular lesion on the lower lip. G) Primary inoculation site with a large crusted lesion. H) Fibrin-covered ulcer on the right tonsil. I) Lesions on the penile glans and foreskin. Source: [27]

In addition to the characteristic rash discussed above, MPOX has other possible clinical features, including systemic illness featuring fevers, chills, and myalgias [21,22]. When systemic symptoms occur, they can present before the rash or shortly after the rash, usually lasting one to five days [23]. This is the traditional array of symptoms common in MPOX until the recent 2022 outbreak, where some patients have manifested without any systemic symptoms. Additionally, some were found to have skin lesions only in the genital, anal, and/or oral regions, which is unique to the 2022 outbreak [24,25]. Genital lesions can present as multiple lesions over the entire genital area or as discrete solitary lesions on the penis [26]. Anal lesions can occur on the buttocks or perianal skin. Many of which cause pain with defecation. Oral lesions are white and circular with a central depression and can be seen on the lips, tongue, or oral mucosa. Localized lesions at the site of infection (anal, oral, or genital) have sometimes been seen to spread to the trunk and face subsequently. It has also been shown that areas contacted during sex often have lesions with the highest viral load, suggesting that close contact is the primary method of transmission during the 2022 outbreak (Figure 2) [24,27]. Also, a quarter of those infected had a concurrent sexually transmitted infection. In patients also infected with HIV, regardless of viral load, skin lesions were found at over three sites in 54% of people compared to 22% of non-HIV infected patients [14].

Spread through the US/World

Spreads of MPOX outside endemic regions of Africa have occurred since the 1970s, as cited above. These spreads are thought to have also occurred due to the eradication of smallpox [3]. The smallpox vaccine eradicated the disease by the 1970s, and that lack of immunity to similar Orthopoxviruses is a plausible reason that MPOX began non-endemic spreads. Since initial case reporting of MPOX began in the 1970s, cases numbered 55 outside of Africa, with 47 cases occurring in the United States between 2000 and 2009, with a majority occurring in 2003 [28]. The 2022 outbreak still has limited data in terms of transmission rates, demographics, and numerical incidences, thus adding an interesting perspective to MPOX non-endemic spreads. In May 2022, two cases of MPOX were reported in the United Kingdom. Surprisingly, these patients had no history of travel to areas of Africa where MPOX is endemic [6]. Since these initial reports were released, thousands of cases have been presented in countries across Europe, South America, North America, and the Middle East. By June 2022, 3000 cases of MPOX had been detected in 47 countries [14]. By the end of July 2022, the WHO had confirmed 16000 cases from 75 territories, and the organization issued a Public Health Emergency of International Concern (PHEIC) [18,29]. The societal implications of this non-endemic MPOX spread are significant to highlight. The goal of this literature review is to provide appropriate, general information about MPOX and its spread and limit discrimination against lesbian, gay, bisexual, and transgender (LGBTQ) communities around the world.

The 2022 outbreak of MPOX occurred during a time of fear, fatigue, and grieving worldwide from the devastating effects of COVID-19. The coronavirus pandemic of 2020 resulted in millions of deaths worldwide and global panic. By the end of July 2021, there were a recorded 4.2 million deaths worldwide from SARS-CoV-2, which causes COVID-19 [30,31]. Quarantines shattered global economies and unemployed millions. In the United States, the political divide regarding the severity of COVID-19 and the efficacy of its vaccines led to public dismay. By May 2022, the sudden outbreak of MPOX led to fears about another pandemic. Seven cases of the West African Clade of MPOX were detected in the US, primarily in transgender men and women, by July 5, 2022 [18,32].

Since these cases consisted of a large number of 20-50-year-old men who were gay, bisexual, or other men who have sex with other men (MSM), it can be investigated that sexual transmission plays a role in MPOX spread [33-35]. Furthermore, studies in Italy and Germany showed patient semen analysis consisting of MPOX [22]. Anal and perineal lesions that contained MPOX involved in sexual contact seemed to also contribute to the sexual transmission of the virus. A 2022 review article on MPOX discussed the societal impact of MPOX spreading among MSM [22,36]. The study calls for an analysis of stigma and discrimination against this group. Discrimination against these individuals occurred in previous outbreaks of the virus, and the Infectious Disease community calls for a push to suppress this discrimination in the current outbreak.

Prevention and treatment

Currently, pre-exposure prophylaxis is not recommended for the general public in the US. However, certain groups of people are at higher risk of contracting the MPOX virus and are eligible for pre-exposure prophylaxis via vaccination [37,38]. According to the CDC, these groups include men who have sex with men, transgender or non-binary people who have been diagnosed with a new STI, or who have more than one sexual partner. These groups also include people who have had sex at a commercial venue in the last six months or in association with a large public event, those with diagnosed HIV, and partners of those in the previously listed groups [37,39]. The live, nonreplicating, modified vaccinia Ankara (MVA) vaccine (also known as JYNNEOS) is the primary vaccine of choice for these groups. However, if not available, the replication-competent smallpox vaccine (ACAM2000) can also be used [9,37,40]. The latter is not preferable due to the higher risk of adverse reactions. The MVA vaccine has an excellent safety profile and is indicated even in immunocompromised persons against MPOX and smallpox with two doses, four weeks apart [37,41,42]. 

Firstly, a patient who tests positive for MPOX needs to be isolated and possibly in a negative pressure room. Patients with MPOX infections who have mild symptoms can recover without medication or an appointment with a provider. These patients can also be managed for their symptoms, with the healthcare provider being cautious of fluid replacement and pain management [43]. For symptomatic management of rash/skin lesions, antihistamines, analgesics, emollients, and topical steroids can be used. The use of systemic steroids in treating more severe symptoms due to MPOX is unclear. However, in rare cases of MPOX leading to sepsis, requiring hemodynamic stabilization, systemic steroids have been used just like in treating other cases of sepsis [44,45]. Antiviral medications may also be used for treatment, but in severe cases. Tecovirimat is the recommended first-line treatment or a combination of tecovirimat and cidofovir [37,43,46]. There are no known drug interactions between tecovirimat and antiretroviral therapy (ART) for HIV infection, making this the first-line treatment option for those co-infected with HIV and MPOX [47]. Tecovirimat inhibits VP37, a viral protein that allows virus particles to infect host cells. Cidofovir is an inhibitor of viral DNA synthesis [43]. Tecovirimat is usually given as a 14-day treatment with contraindications to patients with renal impairment if used intravenously. Cidofovir also has a black box warning for severe nephrotoxicity; therefore, renal function should be monitored in patients on cidofovir [48,49]. Furthermore, teratogenicity and neutropenia have been associated with cidofovir, and WBC counts should also be monitored. Lastly, viral DNA synthesis inhibitor brincidofovir can also be indicated and has fewer side effects than cidofovir, but patients with hepatic impairment and pregnant patients should be closely monitored [50-55].

## Conclusions

MPOX is a zoonotic virus of the Orthopox genus. In 2022, this virus, endemic to Africa, specifically Western Africa and the Congo, spread through multiple countries in Europe, South America, the Middle East, Canada, and the United States. This literature review highlights essential details of the virus and its characteristics. Previous articles regarding spreads, transmission, clinical diagnosis, and treatments of MPOX solidified the groundwork for analysis of the current outbreak. By gathering scientific data and societal reactions to the outbreak, the medical community can provide helpful information, conduct significant research, and aim to educate the public about this disease. In addition, the stigma towards the LGBTQ+ community regarding MPOX and its symptoms and resulting effects should be discussed and attempted to be dismissed. We hope this review can pinpoint important clinical pearls of MPOX and its societal impacts to further promote important discussion of this virus and its disease.
